# DNA Origami Nano-Sheets and Nano-Rods Alter the Orientational Order in a Lyotropic Chromonic Liquid Crystal

**DOI:** 10.3390/nano10091695

**Published:** 2020-08-28

**Authors:** Bingru Zhang, Kevin Martens, Luisa Kneer, Timon Funck, Linh Nguyen, Ricarda Berger, Mihir Dass, Susanne Kempter, Jürgen Schmidtke, Tim Liedl, Heinz-S. Kitzerow

**Affiliations:** 1 Faculty of Science, Department of Chemistry, University of Paderborn, Warburger Straße 100, 33098 Paderborn, Germany; Bingru.Zhang@uni-paderborn.de (B.Z.); juergen.schmidtke@uni-paderborn.de (J.S.); 2 Faculty of Physics, Ludwig-Maximilians-University, Geschwister-Scholl-Platz 1, 80539 Munich, Germany; Kevin.Martens@physik.lmu.de (K.M.); luisa.kneer@physik.lmu.de (L.K.); timon.funck@physik.lmu.de (T.F.); L.Nguyen@campus.lmu.de (L.N.); ricarda.berger@physik.uni-muenchen.de (R.B.); Mihir.Dass@physik.uni-muenchen.de (M.D.); susanne.kempter@physik.lmu.de (S.K.); tim.liedl@physik.lmu.de (T.L.)

**Keywords:** liquid crystals, DNA origami, order parameter, fluorescence dichroism

## Abstract

Rod-like and sheet-like nano-particles made of desoxyribonucleic acid (DNA) fabricated by the DNA origami method (base sequence-controlled self-organized folding of DNA) are dispersed in a lyotropic chromonic liquid crystal made of an aqueous solution of disodium cromoglycate. The respective liquid crystalline nanodispersions are doped with a dichroic fluorescent dye and their orientational order parameter is studied by means of polarized fluorescence spectroscopy. The presence of the nano-particles is found to slightly reduce the orientational order parameter of the nematic mesophase. Nano-rods with a large length/width ratio tend to preserve the orientational order, while more compact stiff nano-rods and especially nano-sheets reduce the order parameter to a larger extent. In spite of the difference between the sizes of the DNA nano-particles and the rod-like columnar aggregates forming the liquid crystal, a similarity between the shapes of the former and the latter seems to be better compatible with the orientational order of the liquid crystal.

## 1. Introduction

Dispersing nano-particles (NPs) in a bulk matrix is a very promising strategy to develop new nanocomposites, i.e., tailored functional materials, which combine or even outperform the properties of their components. If the matrix consists of a liquid crystal (LC) [1,2,3], i.e., an ordered fluid, the positions [4,5,6] or the orientation [7,8,9,10] of the nano-particles may be controlled by the LC structure. Thermotropic and lyotropic LCs are formed by anisometric (for example, rod- or disk-shaped) organic molecules and molecular aggregates, respectively [1,2,3]. In the least ordered phase, the nematic (*N*) phase, these units are preferentially aligned parallel, while their centers of gravity are randomly distributed. The preferred orientation of neighboring molecules or aggregates can be described by a pseudo vector ***n***, the director, which can smoothly vary with position, depending on anchoring conditions and external electric or magnetic fields. The degree of parallel ordering can be described by Zvetkov’s scalar orientational order parameter *S*: = ½ <3 cos^2^θ−1>, where θ is the angle between a molecule or molecular aggregate and the local director, while the brackets indicate ensemble averaging. The order parameter *S* is known to depend on temperature and on the aspect ratio of the units forming the LC. In a LC nanocomposite with low NP densities, rod-like or disk-like nano-particles may locally align parallel or perpendicular to the director ***n***(***r***). At higher concentrations, neighboring particles may also interact with each other, mediated by the complex topology of the distorted director field in their vicinity. The latter effect may lead to the formation of chains or regular lattices of the particles. For completeness, it should be emphasized that these dispersions of nano-particles in a liquid crystalline solvent (as studied in the present work) are different from colloidal liquid crystals, which consist of large fractions of anisometric particles dispersed in an isotropic solvent [11].

The mesogenic behavior of DNA has been frequently studied in different kinds of systems [12,13,14,15,16,17]. Aqueous solutions of DNA can form a lyotropic chromonic liquid crystal (LCLC) [12,13,14], mixtures of DNA and appropriate surfactants can form a thermotropic liquid crystal [16], and nanoparticles of well-defined shape that are fabricated using the DNA origami method can form colloidal liquid crystals, when they are dispersed at high NP concentrations in an isotropic solvent [17,18]. In the present study, a fourth kind of systems is studied, namely a highly diluted dispersion of DNA nanoparticles with well-defined shape in a LCLC, i.e., in an anisotropic solvent. The influence of rod-like or disk-like nano-particles on the orientational order parameter of the LCLC is investigated in the low concentration limit, where interaction of the nano-particles can be neglected. The DNA origami technique [17,18,19,20,21,22,23,24,25,26,27,28,29,30,31,32,33,34,35,36,37,38,39,40] was used in order to synthesize nano-particles with extremely precise shape. In addition to the baseline investigation presented here, LC–NP-composites have potential for complex developments, because the highly precise tailored NPs obtained by the DNA origami technique may be ordered on a larger scale through LC–NP interaction, thereby bridging the gap between NP synthesis and NP assembly, and finally controlling macroscopic material properties. The folding of DNA to three-dimensional nanostructures [20] (DNA origami) is based on combining a single-stranded DNA derived from a natural bacteriophage—the “scaffold” (exhibiting some 7000–8000 nucleotides)—with many shorter single-stranded DNA oligomers—the “staples” (100 to 200 oligomers exhibiting 30 to 50 nucleotides each). Each staple will bind to a specific site of the scaffold and thereby control its folding into a well-defined final shape. This shape can be very complex. The precision of the NP structure is governed by the distance of neighboring nucleotides (≈0.34 nm), the diameter of the DNA double helix (≈2 nm) and the helix pitch (≈10.5 nucleotide spacings). This level of control facilitates fabrication with near-atomic precision [27,28]. Functionalization with chromophores, semiconducting, plasmonic or magnetic particles, and photo- or biosensitive molecular units, tensegrity designs, incorporation of molecular springs or single-stranded DNA handles, and other modifications have not only facilitated the fabrication of very precise static and dynamic nanostructures for microscopic fundamental research, but also made applications in photonics, materials science, biology and medicine feasible [29,30,31,32,33,34,35,36,37,38,39,40].

While previous studies of DNA origami nanostructures were mainly focused on either isotropic solutions or two-dimensional surface assemblies, we here dispersed DNA origami nanostructures uniformly in the bulk liquid crystal solvent:

## 2. Materials and Methods

**Figure 1:**
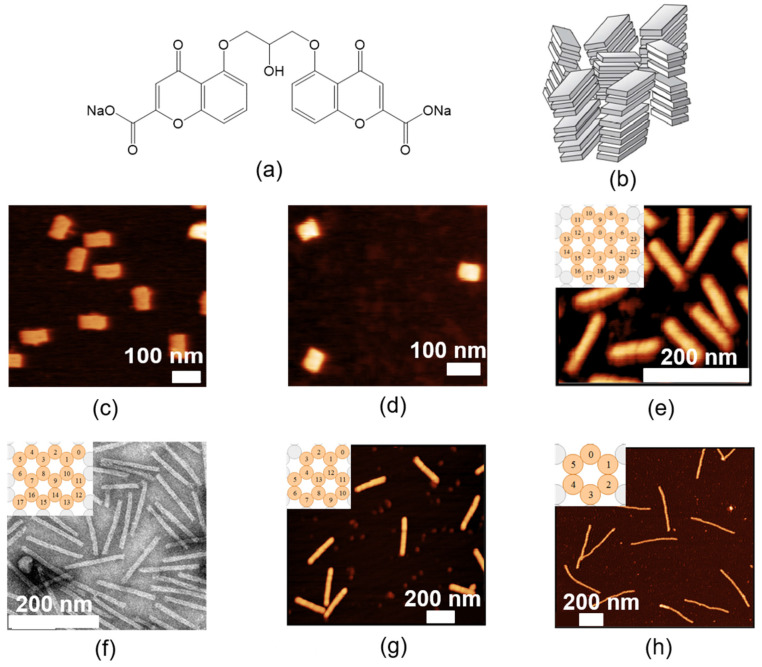
Compounds and nanostructures used in this study. (**a**) Chemical structure of the Disodium cromoglycate (DSCG) molecule. (**b**) Structure of the chromonic *N* phase [41], which consists of columnar molecular aggregates that are orientationally ordered. (**c**–**e**), (**g**,**h**) Atomic force microscope (AFM) images and (**f**) transmission electron microscopy (TEM) image of (**c**) 1LS (layer sheets), (**d**) 2LS, (**e**) 24HB (helix bundles), (**f**) 18HB, (**g**) 14HB and (**h**) 6HB, respectively.

Among the various kinds of LCs, we selected a water-based lyotropic chromonic liquid crystal (LCLC) [41,42,43,44,45,46,47,48,49,50,51,52] for this purpose. The LCLC molecules usually have a plank-like or disk-like polyaromatic central core and two or more ionic groups at the periphery. Disodium cromoglycate (DSCG, Figure 1a) is known as an antiasthmatic drug (“cromolyn”) and is one of the most extensively studied LCLCs. There are two common mesophases of DSCG, the nematic *N* phase (Figure 1b) and the columnar M phase. Both phases consist of columnar molecular stacks with different lengths [41]. So far, there is no experimental evidence on whether the long molecular axes within a single column align strictly parallel to each other (as drawn in Figure 1b) or are randomly distributed perpendicular to the columnar axis. In any case, the *N* phase is known to be optically uniaxial, which might be attributed to the free rotation of the columns along the columnar axis. The aggregation is based on self-organization by noncovalent interaction. In the nematic (*N*) phase, the correlation length of the monomer stacking along the DSCG aggregates axis is approximately 8 nm (≈23 molecules) [48]. Based on earlier experiences with dispersions of colloidal particles in DSCG solutions [53,54,55,56], a nematic solution of 10% (by weight) DSCG (Sigma-Aldrich Chemie GmbH, Steinheim, Germany) in water was doped with DNA nano-particles of different prolate and oblate shapes (Figure 1c–h). Their synthesis followed standard procedures of the DNA origami technique [20,21,22,23,24,25], i.e., a mixture of the DNA scaffold and the DNA staples in an alkaline buffer solution containing 10–18 mM magnesium chloride (MgCl_2_) was first heated to 65 °C and then very slowly cooled to room temperature. Details of the design and the protocol are given in Table 1, in the supplementary material (Figures S1–S6) and in Appendix A, respectively. While the Mg^2+^ cations of MgCl_2_ are known to compensate the repelling interaction of the negative charges of the phosphate groups in DNA very efficiently during the folding process, MgCl_2_ was found in earlier studies to cause precipitation of DSCG [54,55]. Thus, MgCl_2_ had to be replaced by NaCl (Stockmeier Chemie, Bielefeld, Germany) in a solvent exchanging step before mixing the DNA–nano-particle solution with the liquid crystal. Through the atomic force microscope (AFM) test, we confirmed that the shape of DNA origami after dialysis has not changed, and the compact structure has not been unhelixed. The length and width remained the same as before dialysis (Figure S7). The long-term stability of DNA origami nanostructures in NaCl solutions confirms earlier studies [57]. Stable and homogeneous nematic DSCG–NP dispersions were then obtained by mixing a DNA–NP dispersion in 500 mM NaCl solution with an aqueous solution containing 20 wt.% DSCG in a 1:1 ratio.

**Figure 2:**
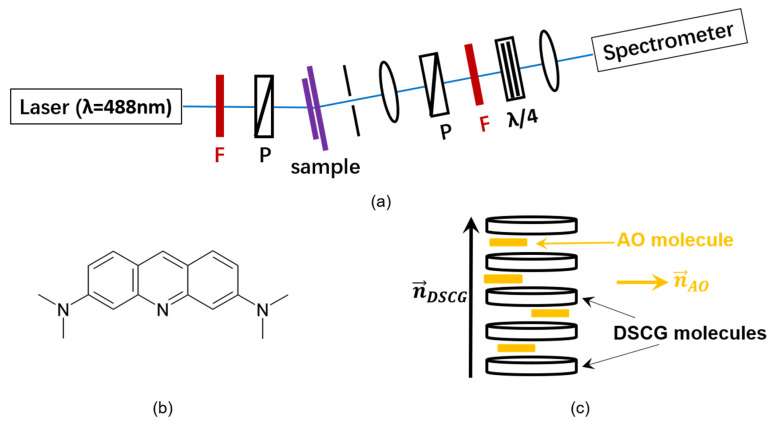
Principles of fluorescence dichroism. (**a**) Experimental setup. F: color filters; P: linear polarizers; λ/4: quarter wave plate. Rotating the polarizers yields the maximum and the minimum fluorescence intensity. (**b**) Chemical structure of an acridine orange (AO) molecule. (**c**) Interaction of the dye with columnar DSCG aggregates.

The scalar orientational order parameter *S* can be measured by studying the linear dichroism. If the transition dipole moments of fluorescent dye molecules show a preferential alignment, the fluorescence emission intensities parallel and perpendicular to this alignment direction are different. In the present work, the setup shown in Figure 2a was used to investigate this effect. For this purpose, the nanocomposites were doped with the dichroic fluorescent dye acridine orange (AO) (Sigma-Aldrich, Munich, Germany) (Figure 2b). The results presented in this paper indicate that AO interacts with DSCG (Figure 2c) so that measuring the order parameter of DSCG is possible by investigating the fluorescence dichroism of DSCG nanocomposites. In principle, AO can also intercalate with DNA [52]. However, the contribution of AO-dyed DNA NPs to the total fluorescence intensity can be neglected if only a small fraction of DNA NPs is present. The very interesting insights obtained from the comparison of DSCG nanocomposites containing DNA NPs of different shape will be explained in the following paragraphs.

## 3. Results

**Figure 3:**
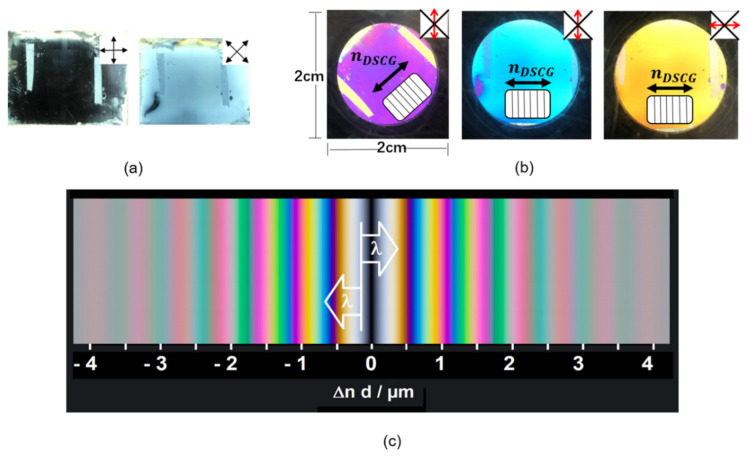
Experimental results indicating uniform alignment and orientational order in chromonic liquid crystal (LC) samples. (**a**,**b**) Polarized optical images of a nanocomposite containing DSCG and DNA nanostructures in an antiparallel rubbed polyimide (PI) cell, (**a**) between crossed polarizers (upper right corner: polarization planes) and (**b**) together with the a λ-retardation plate (slow axis marked red) between crossed polarizers (marked dark). (**c**) Michel–Levy chart of interference colors from birefringent samples.

The nanocomposite containing DSCG and DNA nanostructures was aligned in a sandwich cell coated with polyimide (PI), antiparallel rubbed. If the cell is illuminated with white light and rotated between crossed polarizers, uniformly dark and bright states without interference colors appear, depending on the azimuthal angle of the cell (Figure 3a). The nematic phase shows uniaxial birefringence and the optical axis corresponds to the LC director. Thus, the uniform brightness (Figure 3a) indicates a uniformly planar alignment of the DSCG aggregates. In the dark state, the director is either parallel or perpendicular to the plane of polarization of the incident light. A uniformly bright state appears at an azimuthal angle of 45°. If the sample is aligned at an azimuthal angle of 45° and an additional λ-retardation plate with an azimuthal angle of 45° is inserted, interference colors appear (Figure 3b). These well-known interference colors, which depend on the size of the product Δ*n* *d* of birefringence and layer thickness, can be compared to the Michel–Lévy color chart (Figure 3c). The lack of colors in the bright state that appears at an azimuthal angle of 45° without the λ-plate (Figure 3a) indicates that the retardation between the ordinary and the extraordinary beam is small, within first order, i.e., Δ*n* *d* < λ. If the λ-plate is inserted (Figure 3b), the retardations of the sample and the λ-plate can either compensate each other, so that the total retardation remains within the first order, or add, so that the total retardation is shifted to the second order, as shown in Figure 3c. Since the birefringence of DSCG is known to be negative [44], it can be concluded from this experiment, that the director of DSCG aggregates (***n****_DSCG_*) is horizontal in the samples displayed to the right in Figure 3b.

**Figure 4:**
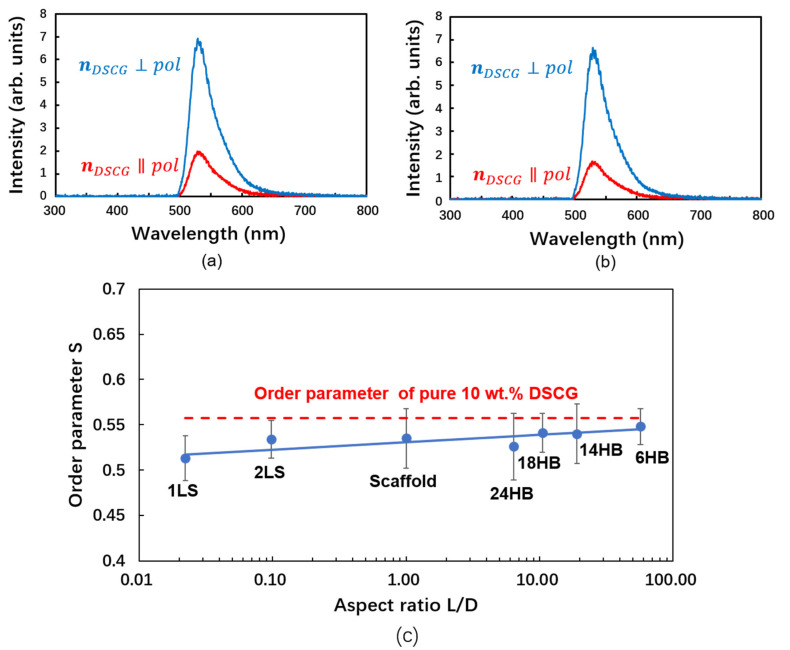
Fluorescence dichroism and order parameter of DSCG solution and respective DNA–NP nanocomposites. (**a**) Polarized fluorescence spectra of 50 µM AO in the presence of 10 wt.% DSCG without any DNA nano-rods. (**b**) Polarized fluorescence spectra of 50 µM AO in the presence of 10 wt.% DSCG and 8.9 nM 24-helix bundles (24HB). The striking similarity of the fluorescence intensities observed for DSCG samples (**a**) without DNA–NPs and (**b**) with DNA–NPs reveals that the presence of small amounts of DNA–NPs does not affect the fluorescence dichroism, which indicates the alignment of the DSCG aggregates. (**c**) Order parameter of DSCG versus aspect ratio of the DNA nanostructures that are added.

For an aqueous solution of 10 wt.% DSCG doped with AO, the fluorescence intensity contribution *I*_⊥_ with polarization perpendicular to the director of DSCG (***n****_DSCG_*) is larger than the intensity contribution *I*_||_ polarized parallel to ***n****_DSCG_* (Figure 4a). This finding indicates that the transition dipole moments, i.e., the long axes of the AO molecules, are preferably aligned perpendicular to ***n****_DSCG_*. The scalar order parameter *S* can be calculated from the fluorescence intensities using the relation *S* = 2 (*I*_⊥_ − *I*_||_)/(2*I*_⊥_ + *I*_||_).

If a small concentration (8.9 nM) of 24-helix bundles (24HB) is dispersed in an aqueous solution of DSCG (10 wt.%) doped with AO (50 µM), the dichroic ratio is slightly reduced, indicating a lower DSCG order parameter (Figure 4b).

In order to explore the relation between orientational order and the shape of the DNA origami nanostructures, *n*-helix bundles (*n*HB, with *n* = 6, 14, 18, and 24) and *m*-layer sheets (*m*LS, with *m* = 1, 2) (Figure 1c–h) showing various length/width ratios *L*/*D* (Table 1) were studied. The final concentrations of DSCG, DNA nanoparticles and NaCl in the final mixture are recorded in Table A2 in Appendix A: Experimental Details. The alignment of the LCLC (10 wt.% DSCG) is found to be reduced by the presence of the nano-particles (Figure 4c). The extent of this reduction varies gradually with the shape of the nano-particles. The orientational order parameter *S* increases with increasing aspect ratio *L*/*D* (Figure 4c, Table 1). This finding indicates that rod-like DNA nano-particles with high aspect ratios are very well compatible with the parallel alignment of the rod-like columnar aggregates of DSCG molecules, which form the nematic phase of the liquid crystalline matrix. In contrast, sheet-like DNA NPs disturb the parallel alignment of the DSCG aggregates and thus reduce the order parameter of the *N* phase of DSCG.

## 4. Discussion

The principal result of the present study, the influence of the NPs on the orientational order of the surrounding nematic LC, can be explained as follows. The DSCG order parameter measured in the present work is a macroscopic average value. Since its local value in a microscopic volume is expected to be constant at constant temperature, the decrease in the average value caused by the presence of NPs can be attributed to deviations of the director from a uniform alignment. The distortion of the nematic director field ***n***(**r**) is known to depend on the competing influences of the elastic properties of the LC and the anchoring of the director at the particle surface. The competing influences of elasticity and anchoring can be described by the extrapolation length ξ: = K/W, where K (unit [*N*]) is an effective mean value of the elastic coefficients and W (unit [J m^−2^]) is the anchoring energy per surface area. A small (large) value of ξ corresponds to a relatively strong (weak) anchoring, which may cause a large (small) distortion of the director field. Colloidal particles with a size much smaller than the extrapolation length do not affect the uniform director alignment, while larger particles are capable of deforming the director field, thereby creating topological defects and increasing the elastic free energy. For nematic DSCG solutions, ξ ≈ 2 µm can be expected [51], i.e., the DNA NPs used in this study are at the weak anchoring limit. In other systems, the alignment of rod-like particles has been investigated for both tangential [9] and perpendicular anchoring [10]. In both cases, the least distortion of the director field was found when the nano-rods aligned along the director [9,10]. In the present case of DNA nano-rods, the observed effects on the average order parameter (Table 1) can qualitatively be explained by the ratio of the diameter of the respective rods to the extrapolation length. Longer nano-rods disturb the director field to a lower extent, because their diameter is much smaller than the extrapolation length. The case of nano-sheets is more complicated. Firstly, the one-layer-sheet is known to be highly flexible and tends to twist [58,59], which may cause more complicated director distortions. Secondly, the alignment of disk-like particles is expected to depend on the surface anchoring. Alignment of the surface normal perpendicular to the director ***n_DSCG_*** is expected for tangential anchoring (as observed in Ref. [9]) and alignment of the surface normal along ***n_DSCG_*** is expected for perpendicular anchoring, respectively. For any case of anchoring, a larger influence of the nano-sheets on the director field is expected than for the nano-rods, because of the large lateral extension of the nano-sheets perpendicular to ***n_DSCG_***. The experimental results described in this article confirm these expectations.

## 5. Conclusions

Complementary to previous studies on LCLCs made of DNA [12,13,14,15], to thermotropic LCs made of DNA [16] and to colloidal suspensions of DNA nanoparticles in an isotropic liquid [17,18], the experiments presented in this paper were focused on diluted suspensions of precisely tailored anisometric DNA nanoparticles in a lyotropic chromonic liquid crystal. The orientational order parameter of the latter is found to be only slightly reduced by the presence of the rod-like nanoparticles and more significantly reduced by sheet-like nanoparticles. These new findings guide the way toward a better understanding and further development of complex nanostructured materials with macroscopic orientational order.

## Figures and Tables

**Table 1:** Order parameter of DSCG samples doped with DNA origami nanoparticles exhibiting different aspect ratios. AFM and TEM images of these DNA origami nanostructures are displayed in Figure 1c,d,e,g,h,f, respectively.

DNA Origami Design	L_th_ (nm)	L_exp_ (nm)	D_th_ (nm)	D_exp_ (nm)	L_th_/D_th_	S
**1LS**	2.00	1.99	89.92	43.88	0.02	0.51
**2LS**	4.60	3.23	47.14	34.70	0.10	0.53
**24HB**	107.10	105.00	16.76	4.66	6.39	0.53
**18HB**	142.80	139.78	13.60	4.46	10.50	0.54
**14HB**	209.68	237.00	11.00	4.09	19.06	0.54
**6HB**	410.78	405.00	7.20	1.58	57.05	0.55

**Figure A1:**
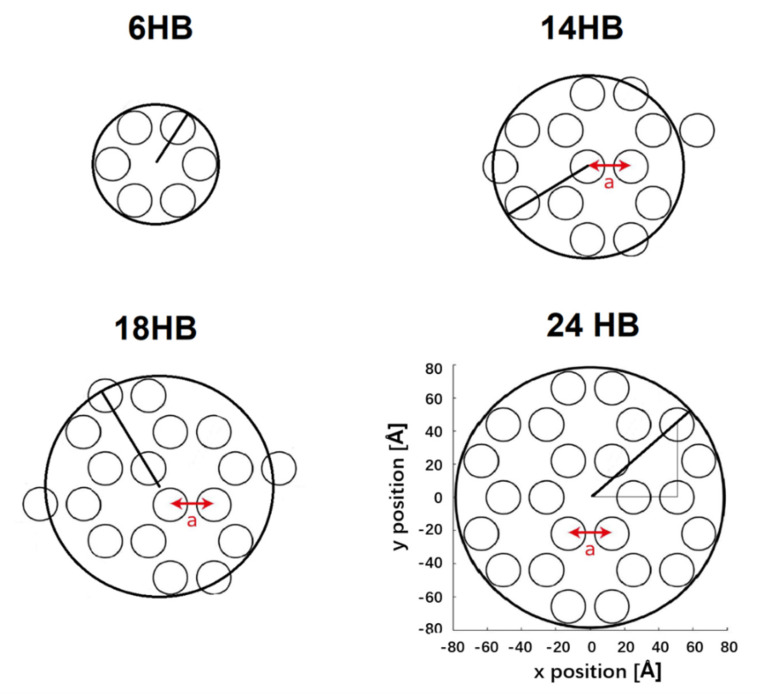
Cross section of DNA origami structure with 6HB, 14Hb, 18HB and 24HB; inter helical distance is a = 2.6 nm calculated with a DNA double strand radius of r = 1 nm and an inter-helical gap of 0.6 nm; Model of 6HB with a radius of R_6HB_ = a + r, 14HB with a radius of R_14HB_ = $\sqrt{3}$ a + r, 18HB with a radius of R_18HB_ = ($\sqrt{3}$ + 1/2) a + r, 24HB with a radius of R_24HB_ = $\sqrt{7}$ a + r.

## Supplementary Materials

The following are available online at https://www.mdpi.com/2079-4991/10/9/1695/s1, Figure S1: Design of the 1-layer sheet (1LS). Top: Cross section. Below: Staple design, Figure S2: Design of the 2-layer sheet (2LS). Top: Cross section. Below: Staple design, Figure S3: Design of the 24-helix bundle (24HB). Top: Cross section. Below: Staple design, Figure S4: Design of the 18-helix bundle (18HB). Top: Cross section. Below: Staple design, Figure S5: Design of the 14-helix bundle (14HB). Top: Cross section. Below: Staple design, Figure S6: Cross section of the 6-helix bundle (6HB) design. The staple design of 6HB is given in Ref. 59, Figure S7: AFM images of (a) 1LS, (b) 2LS, (c) 24HB after dialysis from the folding buffer (1 × TE, 10–18 mM MgCl_2_) to an Mg^2+^-free buffer solution (1 × TE, 0,5 M NaCl). After dialysis, the shape of all origami did not change. The length and width remained the same as before dialysis.

## Appendix A. Experimental Details

### Appendix A.1. DNA Origami Synthesis

Sheet-like and rod-like DNA nanoparticles (*n*-layer sheets, *n*LS with *n* = 1 or 2, and *n*-helix bundles, *n*HB with *n* = 24, 18, 14 and 6) have been assembled from a single stranded DNA scaffold and 170 to 227 single stranded DNA oligomers (staples) (Table A1). The respective folding solution was composed of 5–15 nM scaffold and 50–150 nM staples in TE-/TE-buffer (“Rotistock”, Carl Roth, Karlsruhe, Germany) containing 10–18 mM magnesium chloride (MgCl_2_). Denaturation was carried out in a Thermocycler (Techne, 3Prime, Staffordshire, UK) by heating to 65 °C. Subsequently, the folding mixture was slowly (during 1.5 to more than 24 h) cooled to room temperature in order to allow the folding. Details of the folding process are given in Table A1. The DNA origami structures were tailored by computer aided design using the cadnano [24,60] and the CanDo software [25,26,61] and are presented in the Figures S1 to S6. The staple design of 6HB is given in Ref. [22].

**Table A1:** Experimental details of the DNA nanoparticle synthesis via DNA origami (respective scaffold, number of staples, thermal treatment, buffer and salt concentration used for the folding procedure).

Design	No. of Nucleotides of the Scaffold	No. of Staples	Cooling Duration of Folding (h)	Buffer	Concentration of MgCl_2_ (mM)
**1LS**	7249	184	4.8	TE	16
**2LS**	8064	223	19.8	TE	16
**24HB**	7560	210	25.3	TE	14
**18HB**	7560	196	25.3	TE	18
**14HB**	8634	227	24.6	TE	18
**6HB**	7249 ^1^	170	1.5	TAE	10

^1^ M13mp18 genome scaffold (from Tilibit Nanosystems GmbH, Garching, Germany).

The thickness of the DNA layer sheets and the diameter of helix bundles are calculated assuming a radius r = 1.0 nm for a single double-stranded DNA helix and a spacing of a = 2.6 nm between the centers of two interconnected helices (Figure A1).

After assembly, 1LS, 2LS, 24HB, 18HB and 14HB origami nano-rods were purified by spin filtration (Amicon Ultra 0.5 mL, Ultracel 100 K, Merck, Darmstadt, Germany) and washing with 11 mM MgCl_2_-1× TE buffer to remove excess staple strands. 6HB was centrifugal filtered and washed with water.

The filtered DNA origami structures were immobilized on mica or TEM grids and analyzed by either transmission electron microscopy (TEM), model JEM-1011 (JEOL, Freising, Germany) or atomic force microscopy (AFM, Agilent 5500 und 5100 AFM, intermittent contact mode using silicon cantilevers (HQ:NSC18/Al BS from MikroMasch, Wetzlar, Germany).

### Appendix A.2. Preparation of Liquid Crystal Nanocomposites

Disodium cromoglycate (DSCG) was purchased from Sigma-Aldrich and used as received. It was thoroughly mixed with milli-Q water by repeated heating, agitation, and exposure to ultrasound, until a homogeneous mixture containing 20 wt.% DSCG was obtained.

In earlier studies, it was found that MgCl_2_ can cause a precipitation of DSCG, even with a small concentration [54,55]. In order to avoid this precipitation of DSCG, folded DNA nanoparticles were transferred via dialysis from the folding buffer (1× TE, 10–18 mM MgCl_2_) to an Mg^2+^-free buffer solution (1× TE, 0.5 M NaCl). The concentration of the synthesized DNA nanoparticles was diluted to 20 nM, and this concentration was reconfirmed again using a microvolume spectrophotometer (Nanodrop 2000c) after dilution. After the salt exchange, the respective DNA origami sample was mixed with an aqueous solution of 20 wt.% DSCG in a 1:1 mixing ratio (by volume). The yields of the synthesized 1LS and 2LS are very low, therefore the concentration of these two DNA nanoparticles in the mixture is significantly lower than the others. In order to study the alignment of DNA origami nanoparticles, a dichroic intercalating fluorescent dye, acridine orange (AO), with a concentration of 50 µM was doped in the nanocomposite (Table A2).

**Table A2:** Final concentration of the DNA nanoparticle, DSCG, acridine orange (AO), NaCl and buffer in the mixture.

DNA Nanoparticle	Final Concentration of DNA Nanoparticle [nM]	Final Concentration of DSCG [wt.%]	Final Concentration of AO [μM]	Final Concentration of NaCl [M]	Final Concentration of Buffer
**1LS**	1.81	10	50	0.25	1×
**2LS**	3.88	10	50	0.25	1×
**24HB**	8.90	10	50	0.25	1×
**18HB**	12.00	10	50	0.25	1×
**14HB**	9.63	10	50	0.25	1×
**6HB**	12.28	10	50	0.25	1×

### Appendix A.3. Preparation of Nanocomposite Test Cells

Liquid crystal (LC) test cells were manufactured by two glass substrates coated with antiparallel rubbed polyimide (from the company E.H.C. Co., LTD., Tokyo, Japan). The EHC substrates were used without any cleaning and directly treated in O_2_-plasma (Harrick Plasma Cleaner/Sterilizer model PDC-32G, Ithaca, New York, NY, USA, 10 min excitation time) to completely remove any residues. Two substrates were assembled to a cell using 10 µm Mylar A spacer film. Before assembling the glasses, 20 µL nanocomposite solution was dropped in the middle of the lower substrate surface and then fixed with two-component epoxy glue and clamped with a plastic nylon spring clamp.

### Appendix A.4. Characterization of the Aligned Sample

To characterize the alignment quality of nanocomposite filled cell, two crossed sheet polarizers were used. A spatially uniform brightness (Figure 3a) indicates uniform alignment of the DSCG director. Rotating the sample between crossed polarizers results in a periodic variation of the transmitted light intensity I, indicating planar alignment, where I = I_max_ sin^2^(2φ) is expected (φ being the azimuthal angle of the optical axis, i.e., the director). A first-order retardation λ-plate at φ = 45° was inserted to test the director orientation unambiguously (Figure 3b). For this purpose, the well-known interference colors of uniaxially birefringent samples were studied, which can be found in the well-known Michel–Levy chart (Figure 3c) in textbooks.

### Appendix A.5. Alignment Study of the Dichroic Intercalating Fluorescent Dye

The alignment of acridine orange (AO) was investigated by measuring of their linear dichroism. For dichroic fluorescence measurements (Figure 2a), AO was excited with the linearly polarized beam of an argon-ion laser (wavelength 488 nm). To mimic unpolarized excitation, the cells were rotated by an azimuthal angle of 45° with respect to the polarization plane of the exciting beam. The emission contributions polarized parallel or perpendicular to the director of disodium cromoglycate (DSCG) were selected using a rotatable prism polarizer. These emission contributions were converted into circular polarized waves with opposite handedness by an achromatic quarter wavelength retarder and finally fed via fiber optics to a spectrometer (OceanOptics USB2000+UV-vis-ES, Ostfildern, Germany, spectral resolution 3 nm). The conversion from linear to circular polarization was performed, as the spectrometer had equal sensitivity for left- and right-handed circularly polarized light. All fluorescence experiments were carried out at room temperature (≈22 °C).
