# Supplementary material for: DNA Origami Nano-Sheets and Nano-Rods Alter the Orientational Order in a Lyotropic Chromonic Liquid Crystal
Source: Nanomaterials (Basel). 2020 Aug 28;10(9):1695. doi: 10.3390/nano10091695 (PMC7560128; doi:10.3390/nano10091695)
Supplement: Supplementary file 1 [file nanomaterials-10-01695-s001.pdf]

Supplementary Materials

DNA Origami Nano-Sheets and Nano-rods Alter the Orientational Order in a Lyotropic Chromonic Liquid Crystal

*Bingru Zhang, Kevin Martens, Luisa Kneer, Timon Funck, Linh Nguyen, Ricarda Berger, Mihir Dass, Susanne Kempter, Jürgen Schmidtke, Tim Liedl, and Heinz-S. Kitzerow**

*Email: Heinz.Kitzerow@upb.de

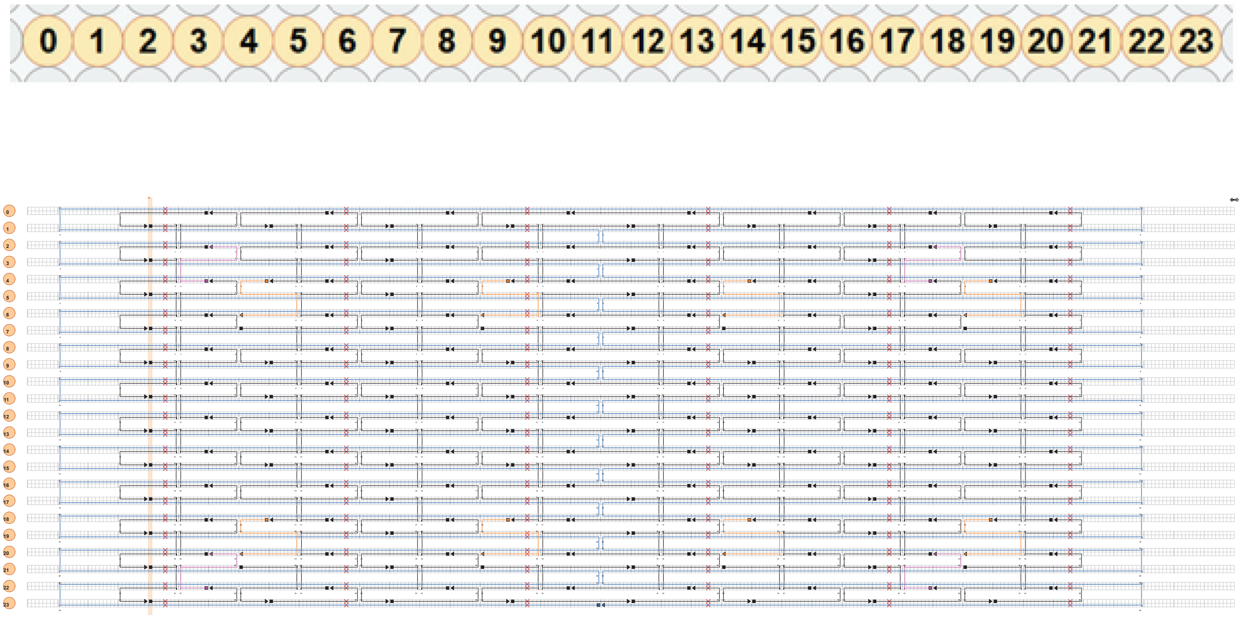

Figure S1. Design of the 1-layer sheet (1LS). Top: Cross section. Below: Staple design.

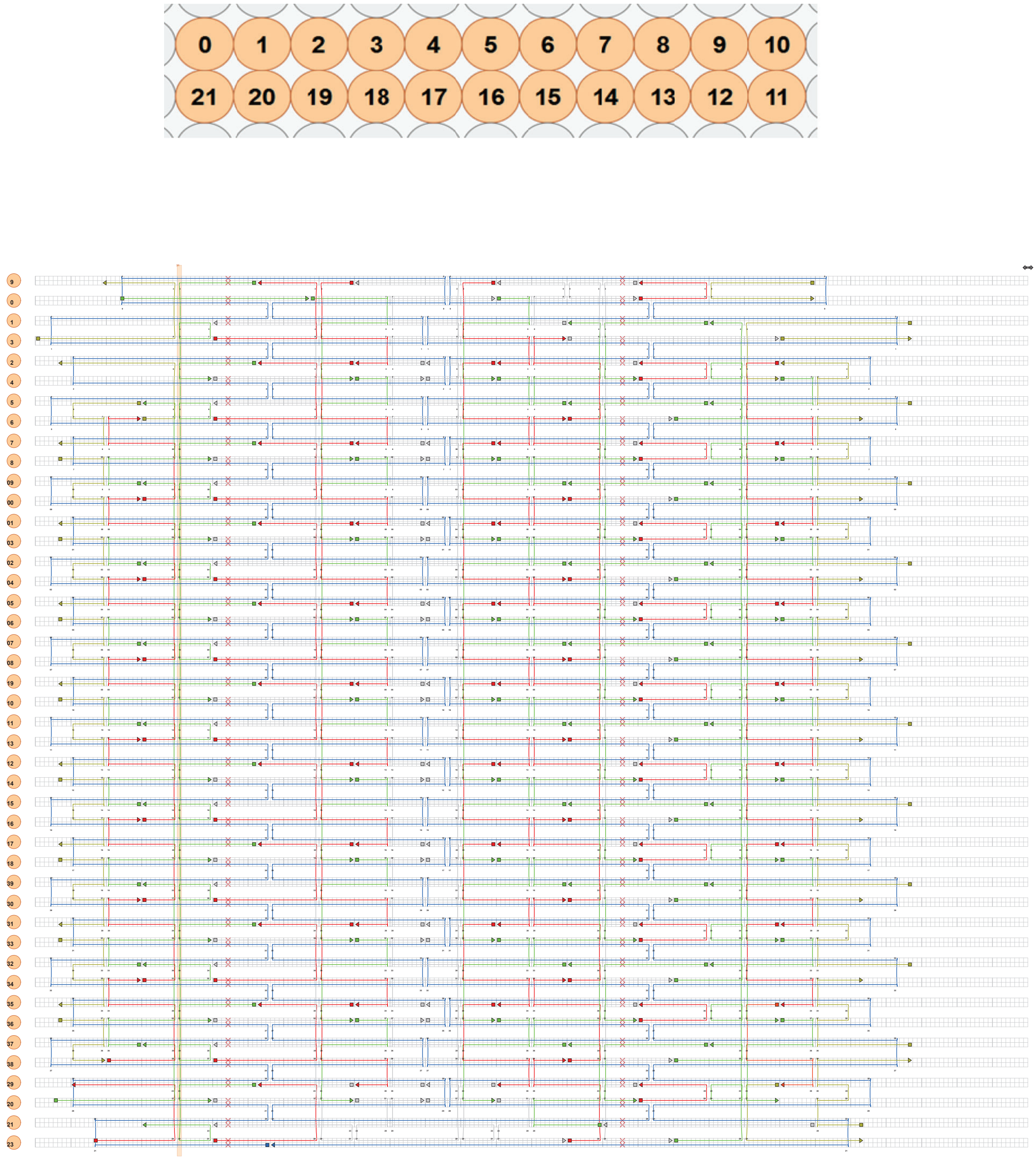

Figure S2. Design of the 2-layer sheet (2LS). Top: Cross section. Below: Staple design.

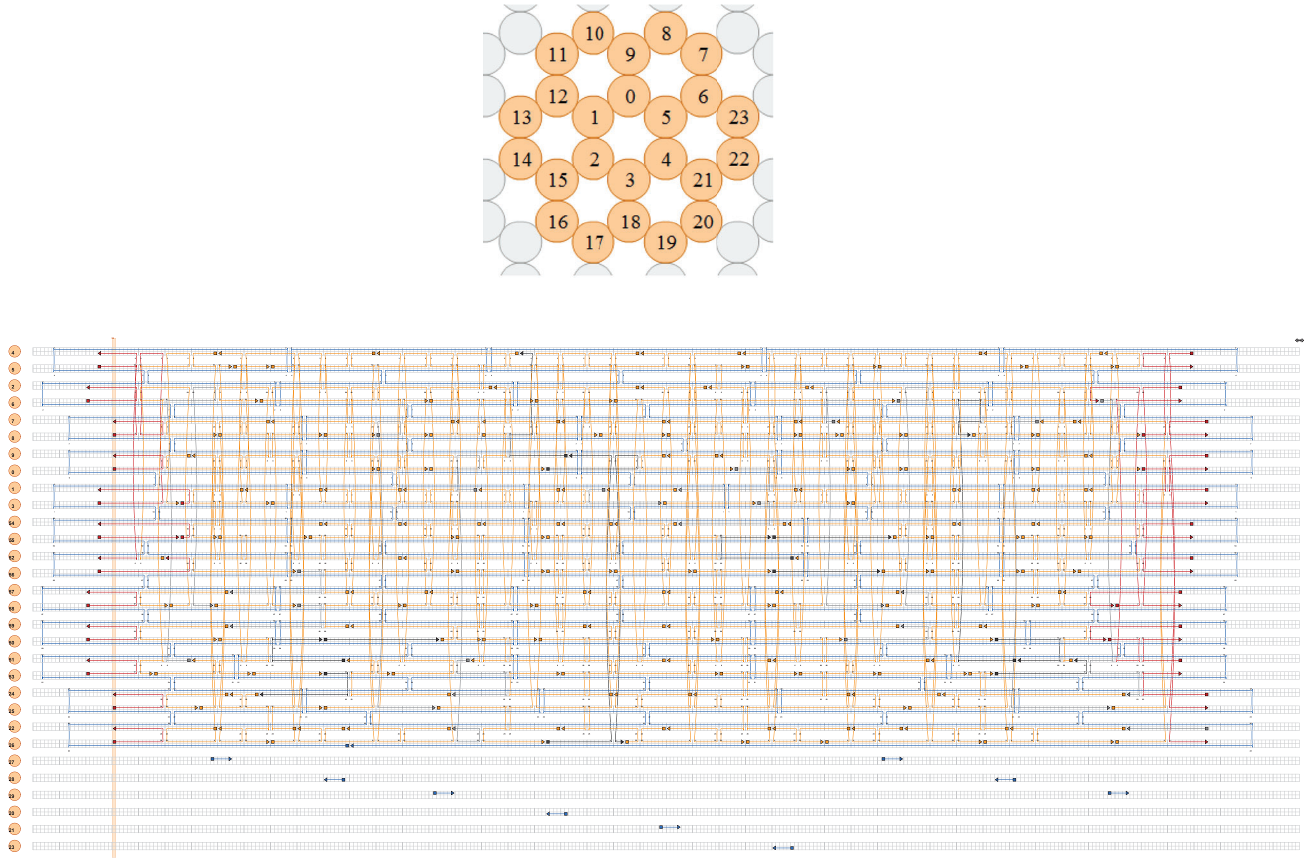

Figure S3. Design of the 24-helix bundle (24HB). Top: Cross section. Below: Staple design.

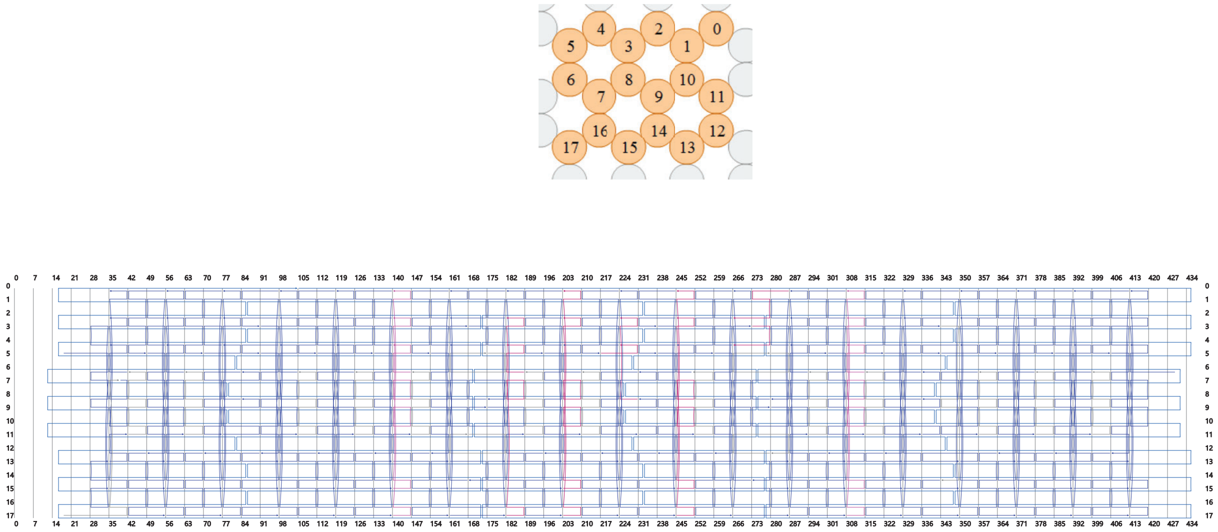

Figure S4. Design of the 18-helix bundle (18HB). Top: Cross section. Below: Staple design.

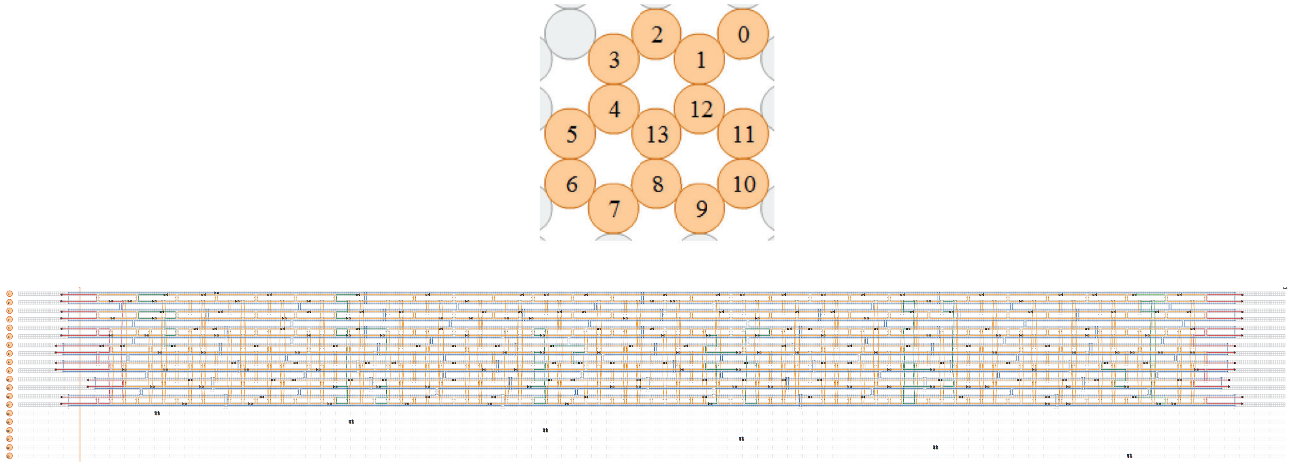

Figure S5. Design of the 14-helix bundle (14HB). Top: Cross section. Below: Staple design.

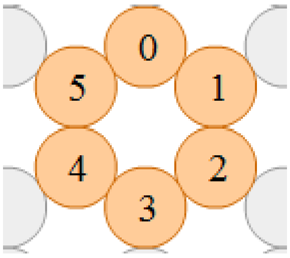

Figure S6. Cross section of the 6-helix bundle (6HB) design. The staple design of 6HB is given in Ref. 64.

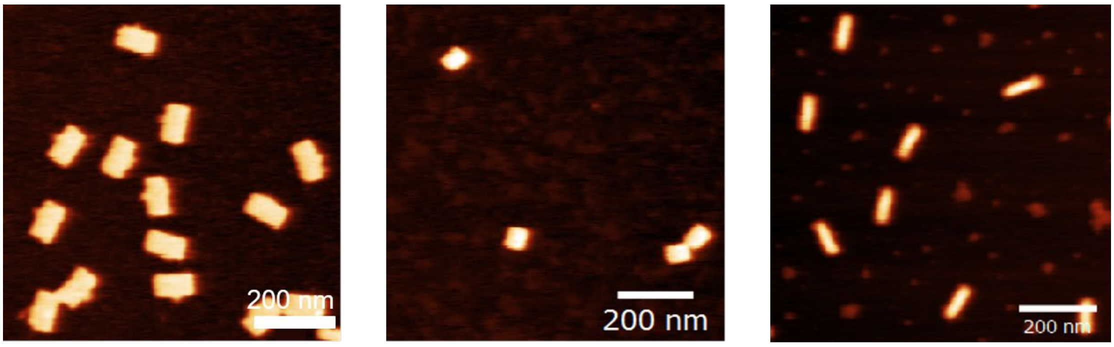

(a) (b) (c)

Figure S7. AFM images of (a) 1LS, (b) 2LS, (c) 24HB after dialysis from the folding buffer (1 x TE, 10 – 18 mM MgCl2) to an Mg2+-free buffer solution (1x TE, 0,5 M NaCl). After dialysis, the shape of all origami did not change. The length and width remain the same as before dialysis.
